# Supplementary material for: Generation and Validation of an Anti-Human PANK3 Mouse Monoclonal Antibody
Source: Biomolecules. 2022 Sep 19;12(9):1323. doi: 10.3390/biom12091323 (PMC9496473; doi:10.3390/biom12091323)
Supplement: Supplementary file 1 [file biomolecules-12-01323-s001.zip › biomolecules-1899773-supplementary.pdf]

Supplemental Figure S1

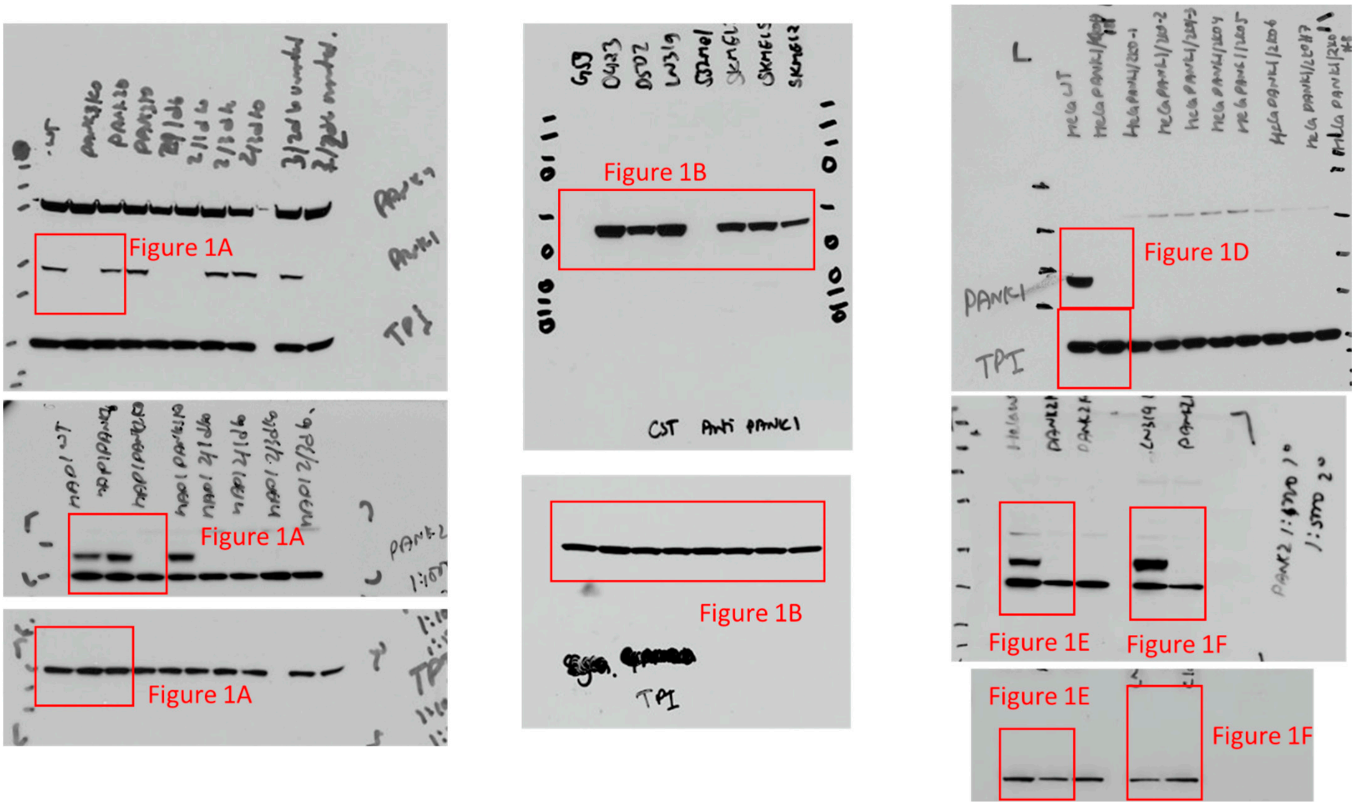

Supplemental Figure S1

Uncropped pictures of the western blots shown in Figure 1. The cropped sections that were used in the figures are highlighted in the red rectangle.

Supplemental Figure S2

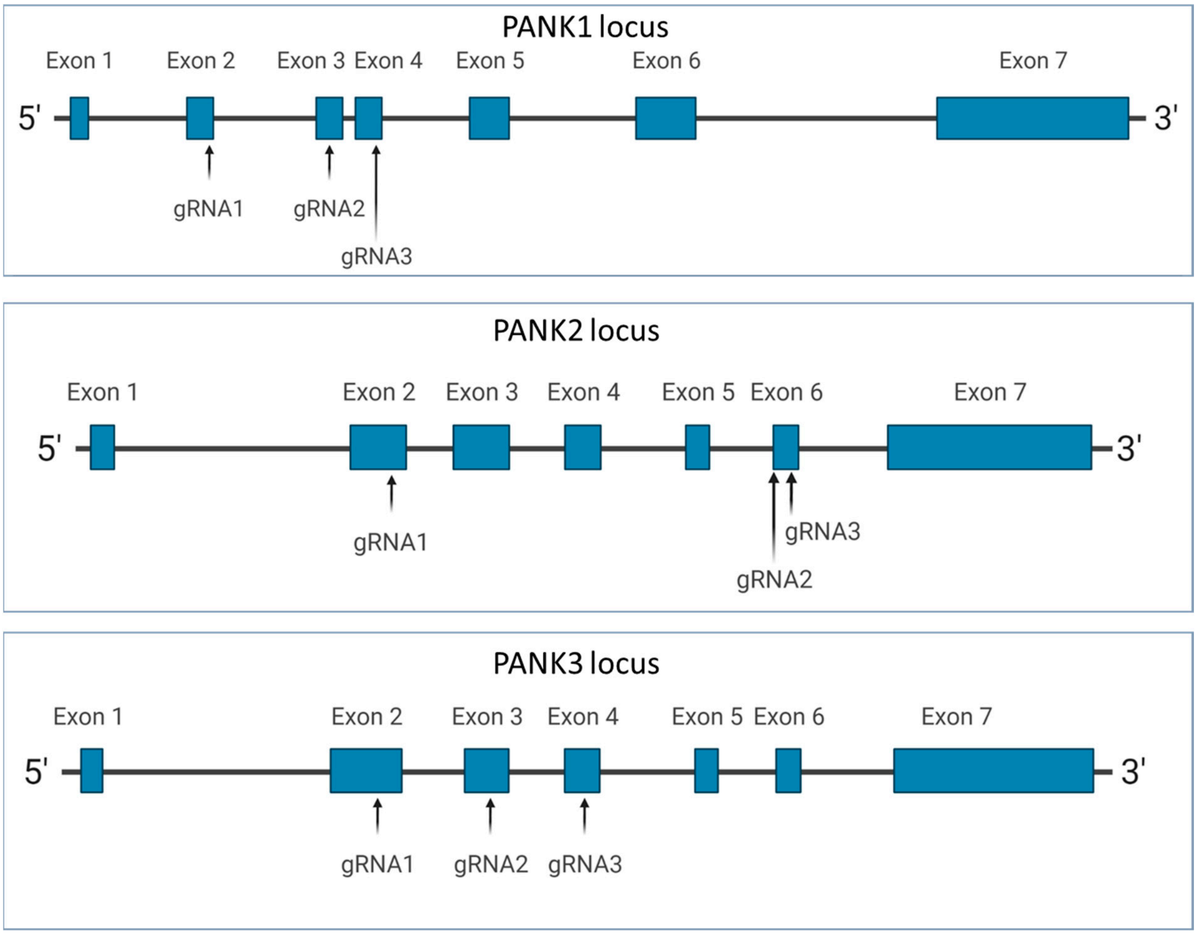

Supplemental Figure S2

PANK1, PANK2 and PANK3 locus identifying 3 different gRNAs that were used to knock the genes out by CRISPR/Cas9 technology.

Supplemental Figure S3

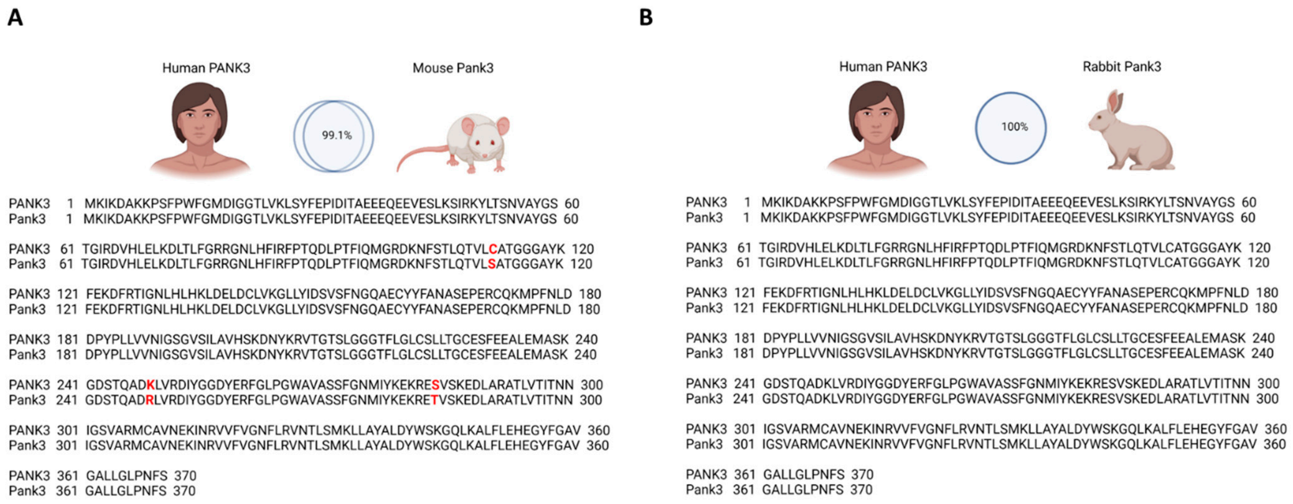

Supplemental Figure S3

Homology of PANK3 protein across species. Amino acid sequence alignment shows that PANK3 protein is highly conserved between humans and rodents. A. Mouse and human PANK3 are 99.1% homologous, while human and rabbit PANK3 proteins are 100% identical.

Supplemental Figure S4

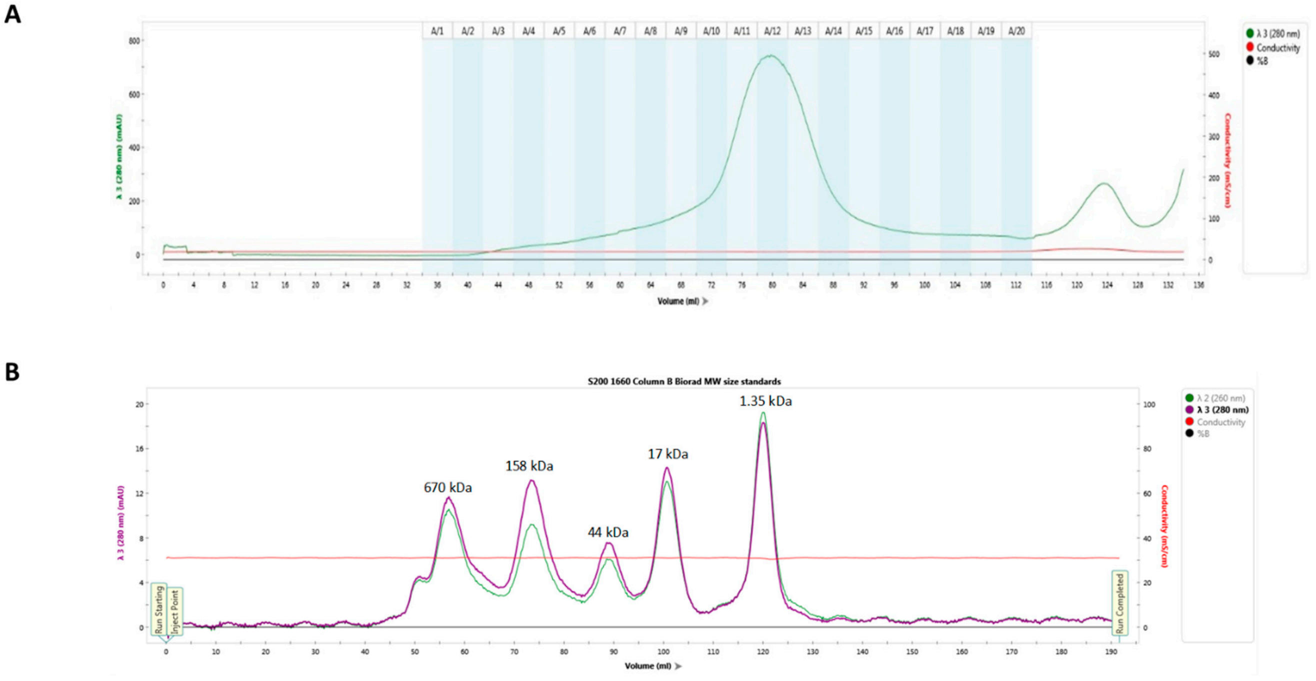

Supplemental Figure S4

Size Exclusion Chromatography elution chromatogram confirms PANK3 protein elutes as homogenous homodimers.

A. Size exclusion chromatography elution chromatogram for PANK3 protein purified using a 120 mL Superdex 200 16/60 column (G.E. life sciences) pre-equilibrated with 20 mM Tris, 200 mM NaCl and 10 mM DTT at pH 8.0. The PANK3 protein elutes as a homodimer at an elution volume of 80 mL. **B.** Elution profile for molecular weight size standard mixture for the Superdex 200 16/60 column showing the elution peaks for Thyroglobulin (670 kDa), γ-globulin (158 kDa), Ovalbumin (44 kDa), Myoglobulin (17 kDa) and Vitamin B12 (1.35 kDa).

Supplemental Figure S5

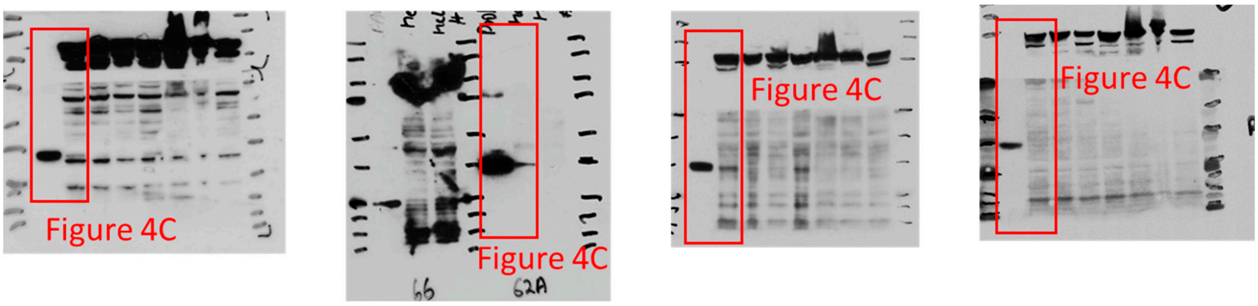

Supplemental Figure S5

Uncropped pictures of the western blots shown in Figure 4. The cropped sections that were used in the figures are highlighted in the red rectangle.

Supplemental Figure S6

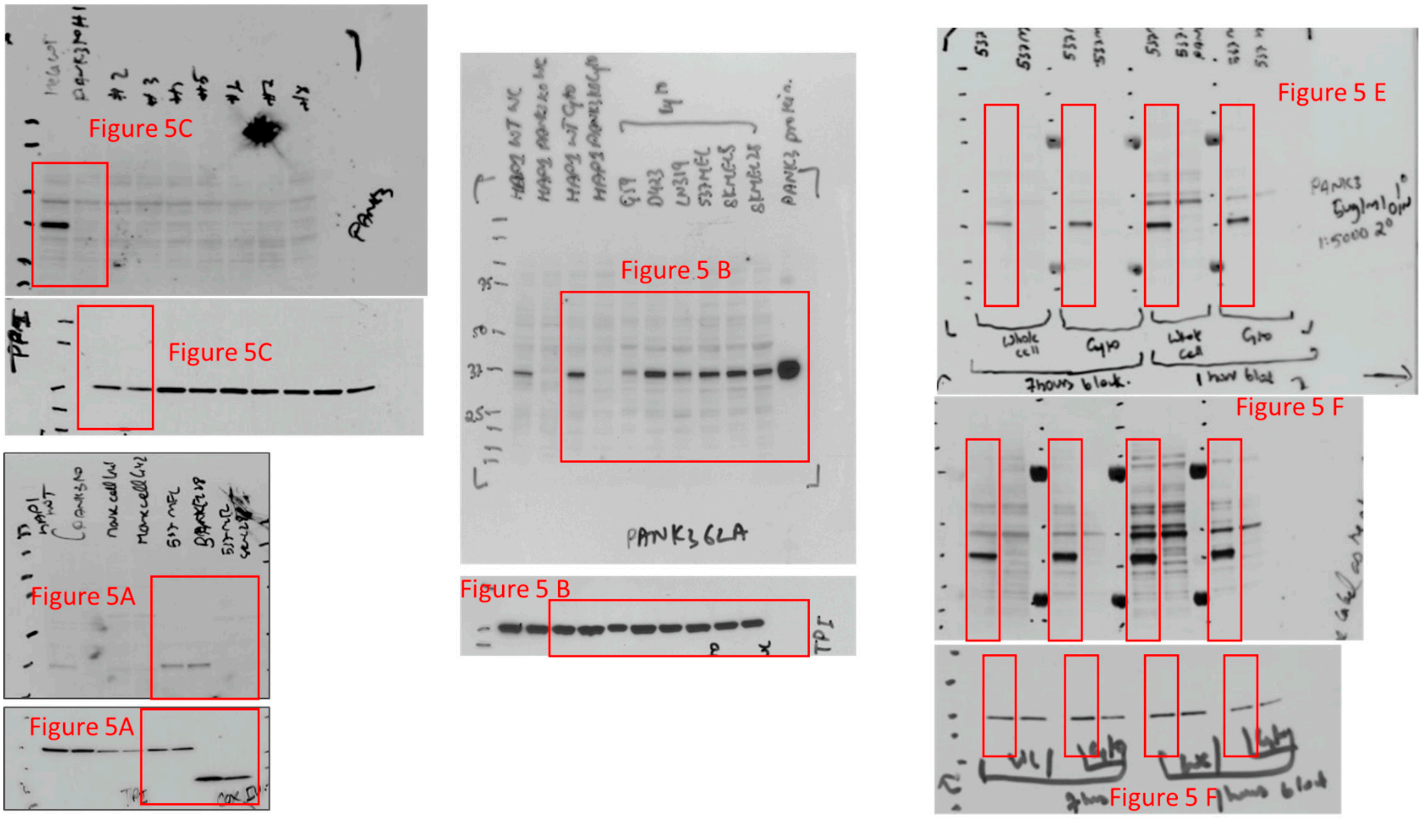

Supplemental Figure S6

Uncropped pictures of the western blots shown in Figure 5. The cropped sections that were used in the figure are highlighted in the red rectangle**.**
